# Effects of Combined Exercise and Calcium/Vitamin D Supplementation on Bone Mineral Density in Postmenopausal Women: A Systematic Review and Meta-Analysis

**DOI:** 10.3390/nu17243866

**Published:** 2025-12-11

**Authors:** Jie Bai, Wenrui Huang, Ruixiang Yan, Xuelian Du

**Affiliations:** 1 School of Nursing and Health, Shenzhen Polytechnic University, Shenzhen 518000, China; 2 The Fourth Clinical Medical College, Guangzhou University of Chinese Medicine, Guangzhou 510405, China; 3 School of Athletic Training, Guangzhou Sport University, Guangzhou 510500, China

**Keywords:** postmenopausal osteoporosis, bone mineral density, exercise, calcium and vitamin D supplementation, meta-analysis

## Abstract

Background: Postmenopausal osteoporosis (PMO) is a major cause of fragility fractures worldwide. While exercise and calcium/vitamin D are standard preventive measures, the synergistic effects of their combined use on bone mineral density (BMD) remain unclear. Methods: We systematically searched eight databases through October 2025 and synthesized data using Review Manager version 5.4. Subgroup, sensitivity, and meta-regression analyses were conducted to examine heterogeneity and test the robustness of results. Risk of bias was assessed using the Cochrane RoB 2.0 tool, and the certainty of evidence was graded with the GRADE framework. Results: 13 RCTs involving postmenopausal women were included. Compared with calcium and vitamin D supplementation alone, combined interventions significantly increased lumbar spine (SMD = 0.31, 95% CI [0.06, 0.55]) and femoral neck BMD (SMD = 0.47, 95% CI [0.09, 0.84]), with consistent but nonsignificant trends at other skeletal sites. Subgroup analyses showed that whole-body vibration produced the greatest and most consistent benefits at both sites, while mind–body or traditional Chinese exercises (e.g., Baduanjin) significantly improved lumbar spine BMD. Shorter interventions (≤6 months) yielded greater gains in BMD, whereas longer durations provided no additional advantage. Conclusions: Exercise combined with calcium and vitamin D supplementation effectively improves bone mineral density in postmenopausal women, especially at the lumbar spine and femoral neck. Whole-body vibration and mind–body exercises show the greatest benefits, with short-term interventions proving most effective. This combined approach offers a practical, evidence-based strategy to preserve skeletal health in aging women.

## 1. Introduction

Postmenopausal osteoporosis (PMO) is a major yet often overlooked global health issue, affecting more than 200 million people worldwide and up to 70% of women over 80 years old [1,2]. The abrupt decline in estrogen after menopause accelerates bone loss, leading to increased fragility and fracture risk; one in three women develops osteoporosis, and their lifetime fracture risk exceeds that of breast cancer [3]. Each year, osteoporotic fractures cause about nine million cases, often resulting in long-term disability or death, with only one-third of patients regaining independence after a hip fracture [2]. Despite advances in diagnosis and treatment, recent data suggest that improvements in mortality have stalled [4]. The economic toll is equally profound, with annual direct costs projected to surpass USD 25 billion in the United States and EUR 47 billion in the European Union by 2030, and nearly 70% spent on treating new fractures while only 5% is allocated to prevention [5]. This reactive, treatment-focused approach underscores a systemic inefficiency and highlights the urgent need to shift toward proactive, preventive strategies. The present study builds on this rationale, evaluating an integrated, non-pharmacological intervention to strengthen bone health and reduce the burden of PMO.

Current international guidelines consistently endorse non-pharmacological strategies, namely exercise and nutritional supplementation, as foundational components of osteoporosis prevention and management [6]. Recommended daily intakes include 1200 mg of calcium and 800–1000 IU of vitamin D [6], alongside regular weight-bearing and resistance exercises [7]. Exercise promotes bone formation, enhances muscle strength and balance, and reduces fall risk, while calcium and vitamin D provide the mineral substrates essential for bone mineralization [8,9,10]. However, these two measures are often implemented independently rather than synergistically, and high-quality evidence regarding their combined effects remains limited. Given their complementary mechanisms, with exercise providing the anabolic “stimulus” and nutrients supplying the “building materials”, a combined approach may achieve greater benefit than either intervention alone [11,12].

Recent studies support this hypothesis. Exercise has been shown to increase bone mineral density (BMD) and favorably modulate bone turnover markers, elevating osteocalcin (OC) and procollagen type I *N*-terminal propeptide (P1NP) levels while reducing *C*-terminal telopeptide of type I collagen (CTX) [13,14]. In contrast, calcium and vitamin D supplementation alone modestly improve mineral balance but show limited effects on fracture risk or BMD [12]. This suggests that nutrient supplementation without sufficient mechanical loading provides material supply but lacks the anabolic drive needed for meaningful bone remodeling. Conversely, combining exercise with calcium and vitamin D may generate a positive feedback loop within the “mechanotransduction–mineralization axis”: mechanical loading triggers bone formation, while optimal mineral and vitamin D status ensures effective matrix mineralization and supports muscle performance [9,10,11]. This multidimensional approach has the potential to simultaneously improve BMD, bone quality, and fall risk, offering a more integrated and cost-effective preventive model.

Building on this rationale, the present systematic review and meta-analysis aims to synthesize available randomized controlled trials to quantify the combined effects of exercise and calcium/vitamin D supplementation on bone health in postmenopausal women. Specifically, it evaluates (1) changes in BMD at the lumbar spine, femoral neck, and total hip; and (2) potential modifiers such as exercise type and intervention duration. By clarifying the magnitude and consistency of these combined effects, this study seeks to provide robust evidence to inform clinical practice and bridge a critical gap in current osteoporosis management guidelines.

## 2. Methods

Following the PRISMA 2020 reporting guidelines [15], this systematic review and meta-analysis were prospectively registered in PROSPERO (CRD420251184502).

### 2.1. Data Sources and Search Strategy

A comprehensive search was conducted in eight databases, including PubMed, Web of Science, Embase, Cochrane Library, China National Knowledge Infrastructure (CNKI), Wanfang Data, VIP Database, and Chinese Biomedical Literature Database (SinoMed). from inception to October 2025 to identify randomized controlled trials (RCTs) evaluating the combined effects of exercise and calcium and/or vitamin D supplementation in postmenopausal women with osteoporosis or low bone mass. The search strategy integrated both keywords and MeSH terms related to “osteoporosis”, “postmenopausal osteoporosis”, “bone mineral density”, “exercise”, “physical activity”, “resistance training”, “calcium”, “vitamin D”, “supplementation”, and “randomized controlled trial” (Supplementary Materials S1). Reference lists of included studies, relevant clinical guidelines, conference abstracts, and trial registries were also manually screened to identify additional eligible studies.

### 2.2. Inclusion and Exclusion Criteria

Eligible studies were RCTs enrolling postmenopausal women diagnosed with osteoporosis or osteopenia based on BMD. Participants were required to be free from secondary causes of osteoporosis, such as endocrine, metabolic, or malignant diseases. Studies were included if the intervention combined structured exercise programs (including weight-bearing, resistance, aerobic, or multimodal training) with calcium and/or vitamin D supplementation, compared with either exercise alone, supplementation alone, or standard care without these interventions. Primary outcomes included changes in BMD at the lumbar spine, femoral neck, or total hip. Exclusion criteria were non-randomized or quasi-randomized designs, studies lacking baseline or outcome data for BMD or bone biomarkers, duplicate publications (with only the most complete or recent retained), and studies involving premenopausal women or patients with secondary osteoporosis, severe comorbidities, or ongoing anti-osteoporotic drug therapy. Reviews, case reports, conference abstracts without full data, and animal or in vitro studies were also excluded.

### 2.3. Data Extraction and Quality Assessment

Two reviewers independently screened all retrieved records, extracted relevant data, and evaluated methodological quality using the Cochrane Risk of Bias 2.0 tool. Extracted information included study characteristics (first author, year of publication, country, and sample size), participant demographics, diagnostic criteria, intervention details (exercise type, frequency, duration, and calcium/vitamin D dosage), control interventions, and follow-up duration. Primary outcome data were recorded for BMD at the lumbar spine, femoral neck, and total hip. Any discrepancies between reviewers were resolved through discussion or consultation with a third investigator.

### 2.4. Certainty of Evidence Assessment

The certainty of the evidence for each outcome was independently assessed using the GRADE (Grading of Recommendations Assessment, Development and Evaluation) approach, as recommended for systematic reviews of randomized controlled trials. This evaluation considered five domains: risk of bias, inconsistency, indirectness, imprecision, and publication bias.

### 2.5. Statistical Analysis

Meta-analyses were performed using Review Manager (RevMan) version 5.4. For continuous outcomes such as BMD, results were expressed as mean differences (MD) with 95% confidence intervals (CI) when units were consistent across studies; otherwise, standardized mean differences (SMD) were applied. Between-study heterogeneity was evaluated using the Chi-square test and *I*^2^ statistic. A fixed-effects model was used when *p* > 0.10 and *I*^2^ < 50%; otherwise, a random-effects model was applied. Sensitivity analyses were conducted by sequentially excluding individual studies to assess the robustness of pooled results. When data permitted, subgroup analyses were conducted by exercise type and intervention duration to explore potential sources of heterogeneity. In addition, sensitivity analyses and meta-regression were performed to assess the robustness of the findings and identify potential covariate influences, while publication bias was evaluated through visual inspection of funnel plots.

## 3. Results

### 3.1. Literature Screening and Included Studies

**Figure 1:**
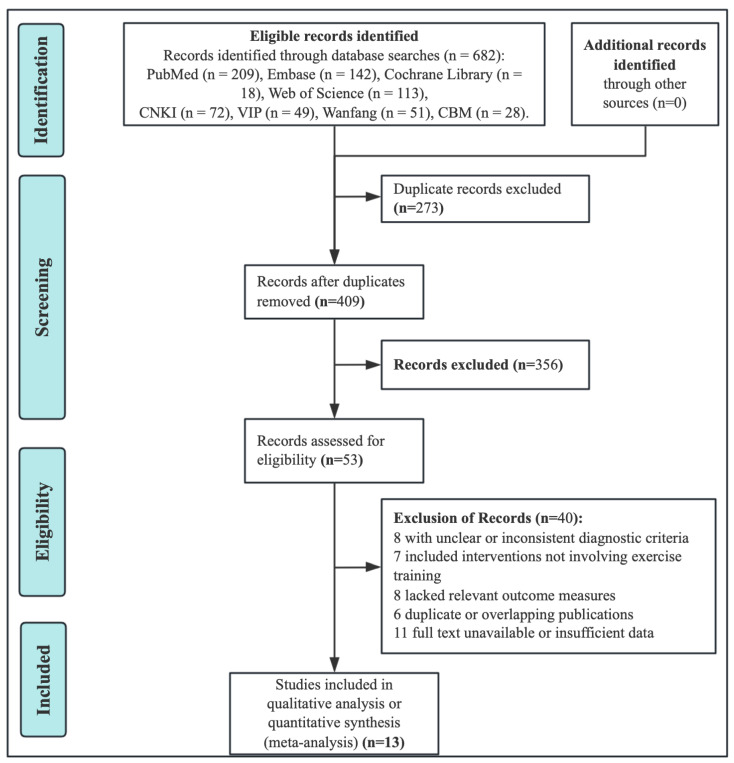
Flowchart showing study retrieval and inclusion.

A total of 682 records were retrieved from eight databases, including PubMed, Web of Science, Embase, Cochrane Library, CNKI, Wanfang Data, VIP Database, and SinoMed. After removing 273 duplicates, 409 studies remained for screening. Based on titles and abstracts, 356 articles were excluded for irrelevance, leaving 53 studies for full-text review. Of these, 40 were excluded due to inconsistent diagnostic criteria, non-exercise interventions, lack of relevant outcomes, duplication, or insufficient data. Finally, 13 RCTs [16,17,18,19,20,21,22,23,24,25,26,27,28] were included in the meta-analysis (Figure 1).

### 3.2. Characteristics of Included Studies

Thirteen studies involving postmenopausal women were included, with sample sizes ranging from 28 to 125 participants and intervention periods from 24 weeks to 24 months. Most trials were randomized controlled studies conducted in China, Japan, Turkey, Egypt, Australia, Sweden, and the United States/France. Participants were generally more than two years postmenopausal, with mean ages between 52 and 68 years. Randomization methods such as sealed envelopes or random number tables were reported in several trials, while a few used assessor or partial blinding. The exercise interventions varied in type, frequency, and intensity, including impact or weight-bearing training, resistance or combined resistance-impact programs, walking or aerobic exercise, traditional mind–body practices (e.g., Baduanjin, square dancing), and whole-body vibration (WBV). Training was typically performed two to seven times per week for 30–75 min per session. Calcium (600–1500 mg/day) and vitamin D (800–1000 IU/day) supplementation were provided in most studies as baseline treatment or control conditions. (see Table 1).

### 3.3. Risk of Bias Assessment

**Figure 2:**
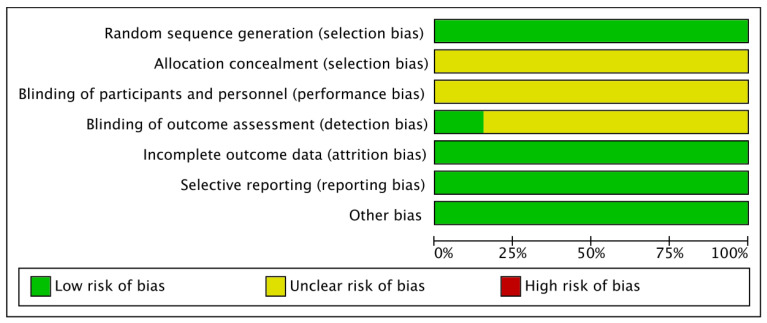
Risk of bias graph.

**Figure 3:**
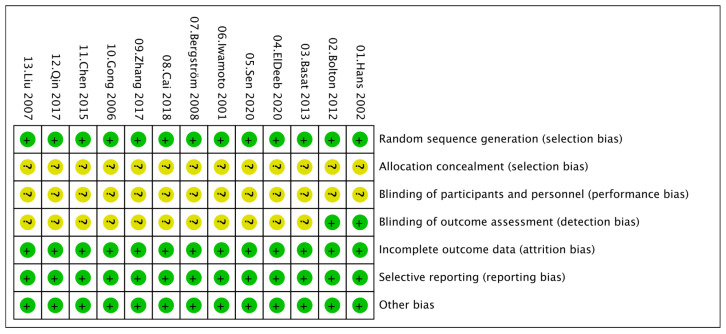
Risk of bias summary.

Overall, the included studies demonstrated a generally low risk of bias. All trials were rated as low risk for incomplete outcome data and selective reporting, indicating adequate follow-up and transparent reporting practices. Most studies accurately described random sequence generation and were assessed as having a low risk in this domain. However, several studies provided insufficient information regarding allocation concealment and blinding, resulting in an unclear risk of bias in these areas. No study was judged to have a high risk of bias in any domain, suggesting that the overall methodological quality of the included trials was acceptable (Figure 2 and Figure 3).

### 3.4. Bone Mineral Density Outcomes

**Figure 4:**
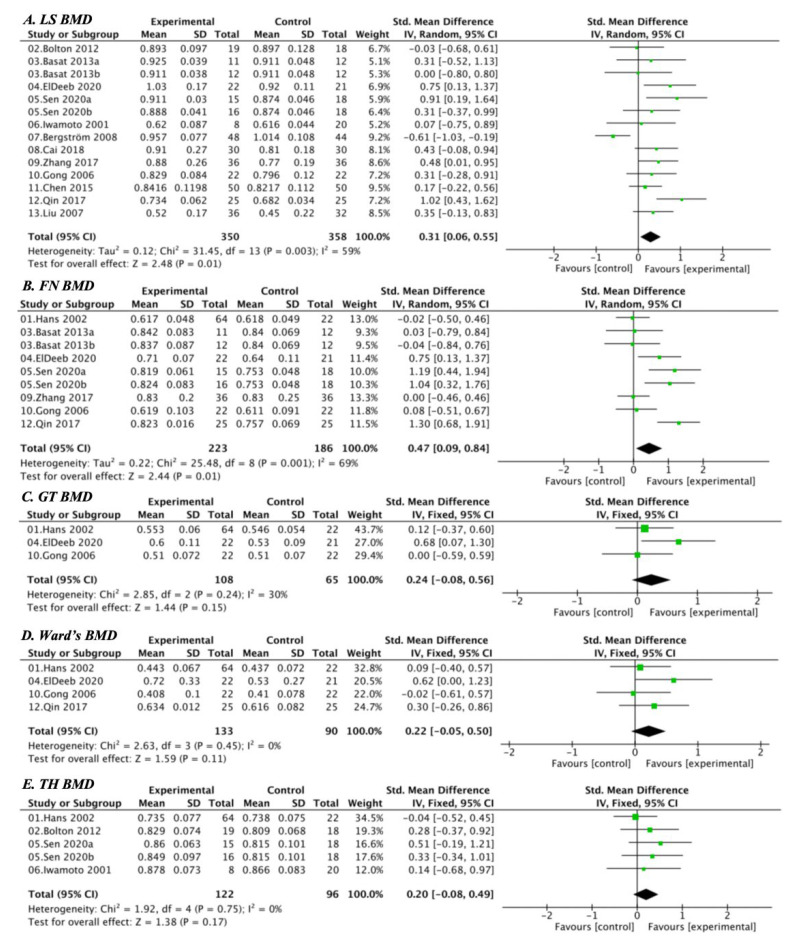
Forest plots comparing BMD outcomes between the exercise + calcium and vitamin D group and the calcium and vitamin D group: (**A**) LS BMD, (**B**) FN BMD, (**C**) GT BMD, (**D**) Ward’s BMD, (**E**) TH BMD.

Five indicators of bone mineral density (BMD) were analyzed, including lumbar spine (LS BMD), femoral neck (FN BMD), greater trochanter (GT BMD), Ward’s triangle (Ward’s BMD), and total hip (TH BMD). Moderate heterogeneity was observed across outcomes (*I*^2^ = 59% for LS BMD, 48% for FN BMD, 30% for GT BMD, 0% for Ward’s BMD, and 0% for TH BMD); therefore, random-effects models were applied for LS and FN BMD, while fixed-effects models were used for the others (Figure 4A–E). Compared with calcium and vitamin D supplementation alone, combined exercise and nutritional intervention significantly increased LS BMD (SMD = 0.31, 95% CI [0.06, 0.55]; *p* = 0.01) and FN BMD (SMD = 0.47, 95% CI [0.09, 0.84]; *p* = 0.02). Although improvements in GT BMD (SMD = 0.24, 95% CI [−0.08, 0.56]), Ward’s BMD (SMD = 0.22, 95% CI [−0.05, 0.50]), and TH BMD (SMD = 0.20, 95% CI [−0.08, 0.49]) did not reach statistical significance, the pooled estimates consistently favored the combined intervention. Overall, these results suggest that exercise combined with calcium and vitamin D supplementation effectively enhances site-specific bone mass, particularly in the lumbar spine and femoral neck of postmenopausal women.

### 3.5. Exercise Type Subgroup Analysis

**Figure 5:**
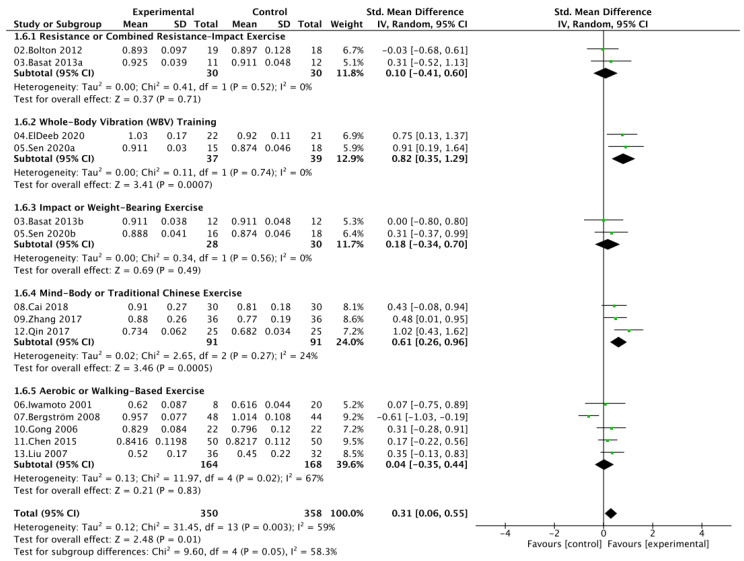
Subgroup analysis of lumbar spine bone mineral density (LS BMD) by exercise type.

**Figure 6:**
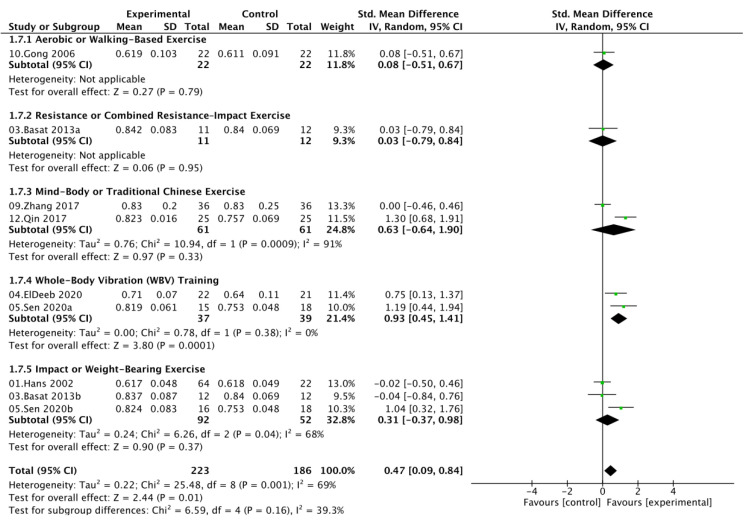
Subgroup analysis of femoral neck bone mineral density (FN BMD) by exercise type.

Subgroup analyses based on exercise type showed distinct effects on bone mineral density across skeletal sites. WBV training produced the most consistent and significant improvements in both LS (SMD = 0.82, 95% CI [0.35, 1.29]) and FN BMD (SMD = 0.93, 95% CI [0.45, 1.41]), with no heterogeneity observed (*I*^2^ = 0%). Mind–body or traditional Chinese exercises, such as Baduanjin or square dancing, also significantly enhanced lumbar spine BMD (SMD = 0.61, 95% CI [0.26, 0.96]; *I*^2^ = 24%) but showed no statistically significant effect on FN BMD, accompanied by considerable heterogeneity. In contrast, resistance or combined resistance-impact training, impact or weight-bearing exercise, and aerobic or walking-based exercise demonstrated nonsignificant effects at either site. Moderate between-subgroup heterogeneity (*I*^2^ ≈ 40–60%) suggested that variations in exercise modality contributed substantially to the observed differences in BMD response. Given the limited number of studies available for GT BMD, Ward’s BMD, and TH BMD, subgroup analyses were not performed to avoid unstable estimates and inflated random error (Figure 5 and Figure 6).

### 3.6. Intervention Duration Subgroup Analysis

**Figure 7:**
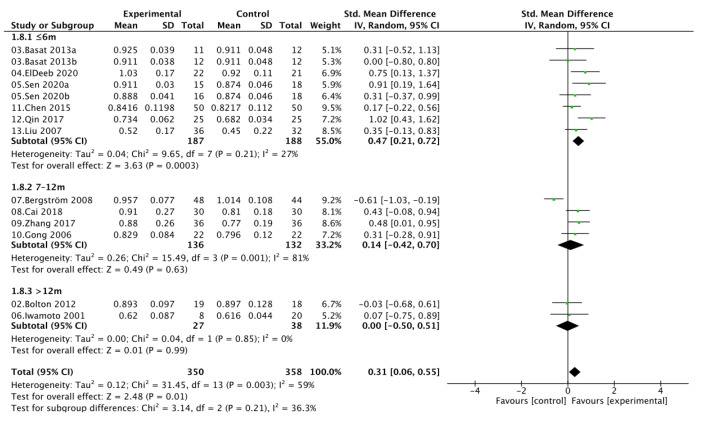
Subgroup analysis of lumbar spine bone mineral density (LS BMD) by intervention duration.

**Figure 8:**
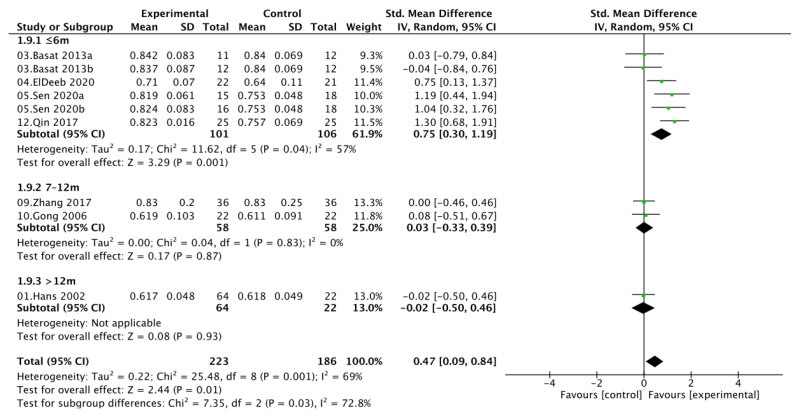
Subgroup analysis of femoral neck bone mineral density (FN BMD) by intervention duration.

Subgroup analyses stratified by intervention duration demonstrated that exercise combined with calcium and vitamin D supplementation yielded time-dependent effects on bone mineral density. For lumbar spine BMD, interventions lasting ≤ 6 months produced significant improvements (SMD = 0.47, 95% CI [0.21, 0.72]; *I*^2^ = 27%), whereas moderate-term (7–12 months) and long-term (>12 months) programs showed attenuated and nonsignificant effects (SMD = 0.14 and 0.00, respectively), with greater heterogeneity observed in the 7–12 month subgroup (*I*^2^ = 81%). A similar pattern was found for femoral neck BMD, where interventions ≤ 6 months led to significant gains (SMD = 0.75, 95% CI [0.30, 1.19]; *I*^2^ = 57%), while those lasting 7–12 months (SMD = 0.03, 95% CI [−0.33, 0.39]) or > 12 months (SMD = −0.02, 95% CI [−0.50, 0.46]) showed no benefit. Between-subgroup heterogeneity was moderate for LS BMD (*I*^2^ = 36.3%) and substantial for FN BMD (*I*^2^ = 72.8%), suggesting that intervention duration may partly explain the variability in treatment response. Collectively, these results indicate that short-term combined exercise-nutrient interventions (<6 months) may be most effective for enhancing bone mass in postmenopausal women, whereas prolonged programs do not confer additional benefit (Figure 7 and Figure 8).

### 3.7. Sensitivity Analysis, Meta Regression, and Evidence Certainty

Sensitivity analyses were performed for all five BMD outcomes. Sequential exclusion of individual studies did not materially alter the pooled effect sizes or the direction of associations, indicating that the results were stable and not driven by any single trial. Although moderate heterogeneity was observed in some outcomes (particularly LS and FN BMD), sensitivity analyses failed to identify any dominant source of variation, suggesting that the overall findings were robust and reliable (Supplementary Materials S2). Meta-regression analyses were conducted to explore potential sources of heterogeneity, including mean participant age, years since menopause, calcium/vitamin D dosage, and geographic region. None of these covariates showed a statistically significant association with the pooled effect sizes for BMD outcomes (*p* > 0.05). These findings indicate that the observed benefits of exercise combined with calcium and vitamin D supplementation were consistent across populations differing in age, menopausal duration, supplementation dose, and study region, suggesting the robustness and generalizability of the intervention effect (Supplementary Materials S3). Visual inspection of the funnel plots for LS and FN BMD revealed a generally symmetrical distribution of effect sizes, indicating no apparent publication bias (Supplementary Materials S4). In addition, the certainty of evidence for all BMD outcomes, as evaluated using the GRADE approach, was rated as moderate, reflecting generally consistent findings across studies with only minor concerns regarding risk of bias and heterogeneity. This indicates that the observed benefits are supported by reasonably robust evidence, providing moderate confidence in the reliability and clinical applicability of the results (Supplementary Materials S5).

## 4. Discussion

### 4.1. Primary Finding

This meta-analysis provides comprehensive evidence that combining exercise with calcium and vitamin D supplementation effectively improves bone mineral density in postmenopausal women, with the most pronounced effects observed at the lumbar spine and femoral neck. Among different exercise modalities, whole-body vibration yielded the most consistent benefits, while mind–body or traditional Chinese exercises such as Baduanjin also enhanced lumbar spine bone mass. Resistance, impact, and aerobic training showed limited effects. The analysis further revealed that shorter intervention durations (within six months) were associated with greater gains in bone density, suggesting an early but potentially transient responsiveness to combined interventions. Sensitivity and meta-regression analyses confirmed the robustness of these findings, showing no single study or covariate-such as age, menopausal duration, supplement dose, or region-significantly influenced the pooled estimates. Overall, these results underscore the value of structured exercise integrated with adequate nutritional support as a feasible and generalizable strategy to preserve skeletal health in postmenopausal women.

### 4.2. Comparison with Previous Studies

This systematic review and meta-analysis provide strong evidence that structured exercise, combined with calcium and vitamin D supplementation, significantly improves BMD in postmenopausal women-particularly in trabecular-rich regions, such as the lumbar spine and femoral neck. These findings not only confirm the superiority of combined intervention but also challenge the traditional hierarchy of exercise modalities.

Our analysis demonstrates that combining exercise with calcium/vitamin D supplementation is substantially more effective than either approach alone, supporting the need for an integrated strategy. Previous large-scale meta-analyses have shown that calcium or vitamin D supplementation alone produces minimal or no improvement in BMD at the LS or FN and has little effect on fracture risk [12]. While supplementation can correct serum 25(OH)D deficiency [9], it does not stimulate bone formation in the absence of an anabolic signal such as exercise. Exercise, on the other hand, provides the necessary mechanical stimulus to activate cellular bone remodeling pathways [3,29]. However, its benefits may be limited when calcium or vitamin D levels are suboptimal, as commonly seen in postmenopausal women. In this context, exercise delivers the mechanical signal, while supplementation provides the biochemical substrate, forming a complementary “signal-plus-substrate” model of bone formation [3]. The superior effect of the combined intervention arises precisely from this physiological synergy.

This study also highlights a critical reordering of exercise effectiveness: WBV and mind–body exercises (such as Baduanjin and Tai Chi) outperform traditional resistance and aerobic training. Multiple meta-analyses have confirmed that WBV significantly increases LS and FN BMD [30,31], likely through its ability to generate high-frequency, low-magnitude mechanical stimuli that enhance osteocyte activation. Similarly, low-intensity mind–body exercises like Baduanjin and Tai Chi have been shown to improve LS BMD effectively [30,32,33]. These findings suggest that bone adaptation depends not only on load magnitude but also on signal frequency, complexity, and duration [34].

In contrast, while resistance training (RT) is theoretically a strong osteogenic stimulus, its real-world effect is often blunted by suboptimal implementation. Protocols using less than 70% of one-repetition maximum (1 RM) or performed fewer than three times per week show markedly reduced benefits [35]. Therefore, the “limited effect” observed for RT likely reflects insufficient training dose rather than true ineffectiveness. Aerobic exercise, such as walking or swimming, provides minimal skeletal loading and is not expected to meaningfully increase BMD [36], consistent with prior evidence.

Although these results may seem unexpected, whole-body vibration and mind–body exercises are not truly “static” in their physiological effects. Whole-body vibration delivers rapid, low-intensity mechanical signals that stimulate bone cells even with minimal joint loading. Mind–body practices such as Baduanjin also provide meaningful mechanical input through sustained muscle engagement, controlled postural shifts, and steady core activation, creating a continuous stimulus for bone adaptation. In contrast, many resistance or weight-bearing programs in the included trials were performed at relatively low intensity or with limited progression. Such programs often fall below the level of mechanical loading needed to produce measurable gains in bone mass. Taken together, the stronger effects observed for vibration and mind–body exercises likely reflect differences in the quality of the mechanical stimulus delivered, rather than the traditional classification of these activities as “static” or “non–weight-bearing.”

### 4.3. Possible Mechanistic Explanations

#### 4.3.1. Dual-Signal Hypothesis: Interaction Between Mechanical Loading and Biochemical Availability

The superior effect of the combined intervention can be attributed to the interaction between mechanical signaling and biochemical substrate availability. Exercise induces mechanical loading that activates mechanotransduction pathways in osteocytes, particularly the Wnt/β-catenin signaling cascade, promoting osteoblast differentiation while suppressing osteoclast formation [37,38,39,40,41,42]. Meanwhile, calcium and vitamin D ensure adequate bone mineralization and calcium homeostasis, with vitamin D acting through its receptor to regulate the balance between bone formation and resorption. Importantly, vitamin D also enhances muscle strength and function [10,43], generating greater contractile forces during exercise. These stronger mechanical loads amplify the osteocytic response, creating a positive feedback loop among muscle, bone, and calcium/vitamin D metabolism—thereby reinforcing both the anabolic and mineralization processes.

Beyond these nutrients, dietary protein is also an important modulator of the muscle–bone axis. Protein provides essential amino acids for collagen synthesis, stimulates IGF-1 production, and supports muscle strength, all of which reinforce osteogenic responses to mechanical loading. Unmeasured variability in habitual protein intake across trials may therefore have influenced participants’ responsiveness to exercise and supplementation [44].

#### 4.3.2. Bidirectional Mechanism of WBV

The consistent benefit of WBV may result from its dual mechanism of action-stimulating bone formation while simultaneously reducing bone resorption. High-frequency, low-magnitude vibrations produce fluid shear stress within bone canaliculi, directly stimulating osteocytes [45] and activating the Wnt/β-catenin pathway to promote osteogenesis. At the same time, WBV lowers the RANKL/OPG ratio, thereby suppressing osteoclast differentiation and activity [45]. This dual effect enables WBV not only to induce new bone formation but also to counteract the excessive bone resorption characteristic of postmenopausal osteoporosis, achieving a net gain in bone mass through both pathways.

#### 4.3.3. Time-Dependent Effects and the “Bone Remodeling Transient”

Our meta-analysis found that interventions lasting six months or less produced greater increases in BMD, consistent with the classical bone remodeling transient model [46]. In early phases of treatment, enhanced calcium and vitamin D availability reduces the initiation of new resorption sites, while existing bone formation continues unabated-resulting in a temporary net gain in bone mass [46]. Supporting biochemical evidence shows that, within six months of intervention, CTX levels decrease while osteocalcin levels rise, reflecting reduced resorption and increased formation [14]. This transient “bone-filling” effect explains the pronounced short-term improvements observed in several studies [47].

In addition, several factors may explain why short-term interventions appeared more effective than longer programs. First, the bone remodeling transient predicts an early, disproportionate gain in BMD due to the rapid suppression of bone resorption and delayed coupling of bone formation; once remodeling equilibrium is re-established after approximately 6–12 months, further increases in BMD naturally plateau [48]. Second, adherence typically declines over time in exercise-based RCTs, and reductions in training frequency, intensity, or supplement compliance can attenuate long-term effects, leading to an apparent loss of efficacy in extended programs. Third, longer trials often accumulate more variability, including seasonal changes in physical activity, intercurrent illness, and fluctuations in dietary or lifestyle factors, which can dilute true BMD changes when measurements are taken at annual intervals [49]. To our knowledge, current clinical guidelines recommend long-term physical activity for musculoskeletal health, but do not explicitly specify a time-pattern of BMD gains (i.e., early gains + plateau). Our data thus provide novel insight into the temporal dynamics of BMD response.

### 4.4. Clinical Implications

This study underscores the need to move beyond the general recommendation of “exercise more” toward a precision-based exercise prescription specifically targeting bone health. Although current clinical guidelines (such as those from the American Association of Clinical Endocrinologists, AACE) recommend calcium and vitamin D supplementation alongside weight-bearing and balance exercises [50,51], they lack specificity regarding which exercise types provide the greatest skeletal benefits. Our findings clarify this gap. Both WBV and Baduanjin emerged as highly effective, safe, and feasible interventions with excellent adherence rates. In practice, healthy and physically capable women can benefit most from high-intensity resistance training (≥70% 1 RM) [52], while frail, mobility-limited, or pain-prone individuals should not view WBV or Baduanjin as “second-best” options. Instead, these are validated primary interventions supported by robust evidence for improving BMD safely and sustainably [53,54,55]. Sensitivity and meta-regression analyses further revealed that age, years since menopause, supplement dosage, and geographic factors did not significantly modify the observed effects. This absence of effect modification strengthens the universality of our findings: the synergy between exercise and supplementation appears to be a fundamental physiological mechanism consistent across populations and settings [29]. From a practical standpoint, clinicians should also consider integrating adequate dietary protein intake into lifestyle prescriptions. Emerging evidence suggests that appropriate protein consumption supports BMD and may enhance the skeletal benefits of physical activity, whereas very high protein intake without adequate activity may exert neutral or even adverse effects [44,56]. Therefore, these results carry substantial implications for clinical translation and public health practice.

### 4.5. Strengths and Limitations

This meta-analysis possesses several notable strengths. First, it represents the most comprehensive synthesis to date evaluating the combined effects of structured exercise and calcium/vitamin D supplementation on BMD in postmenopausal women. The study followed PRISMA and Cochrane methodological standards, incorporating exclusively randomized controlled trials with clearly defined intervention protocols and follow-up durations, thereby enhancing internal validity. Second, the analysis distinguished exercise modalities and intervention durations through predefined subgroup and meta-regression analyses, allowing mechanistic interpretation rather than simple pooled effects. Third, the robustness of findings was confirmed through multiple sensitivity analyses, with consistent results across skeletal sites and no single study exerting undue influence. Finally, publication bias was minimal, and between-study heterogeneity was partially explained by exercise type and intervention duration, indicating the stability and reliability of the overall conclusions.

Despite the robustness of results, several limitations should be acknowledged. First, substantial heterogeneity existed in resistance and combined training protocols-variations in frequency, load progression, and supervision intensity may have diluted the true effects of specific modalities. Second, adherence data were incompletely reported in many trials, which limits the interpretation of real-world effectiveness. Third, BMD serves as a surrogate endpoint and may not fully capture reductions in fracture risk. Fourth, regional and methodological diversity-particularly among Asian studies employing mind–body exercises such as Baduanjin-may affect generalizability. Lastly, the sample size for certain skeletal sites (e.g., Ward’s triangle, total hip) remained limited, restricting the precision of subgroup estimates.

Importantly, none of the included trials systematically assessed background dietary intake, particularly total protein consumption. Dietary protein is an established determinant of bone metabolism, influencing collagen formation, IGF-1 production, calcium absorption, and muscle strength. Both review and population-level evidence indicate that protein intake shows complex interactions with physical activity and calcium/vitamin D status, affecting BMD in a nonlinear and threshold-dependent manner. The absence of dietary data therefore introduces residual confounding and may partly contribute to between-study variability in effect sizes.

Future research should prioritize several directions: ① Head-to-head RCTs comparing high-intensity resistance training, whole-body vibration, and Baduanjin under standardized supplementation protocols; ② Mechanistic investigations elucidating how mind–body exercises trigger osteogenic responses-whether through sustained core tension, neuromuscular activation frequency, or hormonal modulation; ③ Long-term follow-up studies assessing whether short-term BMD gains translate into fracture risk reduction; and ④ Cost-effectiveness and adherence analyses contrasting WBV equipment-based interventions with scalable community programs like Baduanjin or square dancing.

Collectively, this work provides high-level evidence supporting the integration of structured exercise and nutritional supplementation in postmenopausal osteoporosis management, while also identifying methodological gaps that warrant rigorously designed future trials.

## 5. Conclusions

This meta-analysis demonstrates that exercise combined with calcium and vitamin D supplementation significantly enhances bone mineral density in postmenopausal women, particularly at the lumbar spine and femoral neck. Whole-body vibration and traditional mind–body exercises such as Baduanjin appear most effective, while short-term interventions (<6 months) yield the greatest gains. These findings highlight that appropriately structured exercise, when integrated with adequate nutritional support, can serve as a practical and evidence-based strategy for maintaining skeletal health in aging women. Future research should explore optimal exercise intensity, long-term sustainability, and the translation of bone density improvements into fracture risk reduction.

## Figures and Tables

**Table 1:** Baseline characteristics of included studies.

Study	Country	Sample Size (T/C)	Duration	Menopausal Status (yrs)	Age (T/C)	Intervention	Frequency	Control	Supplementation
Hans 2002 [16]	USA, France	99/26	24 mo	≥5	67.6 ± 5.2/66.3 ± 4.9	Heel-drop weight-bearing exercise	120 reps/day, 3–5 min	Ca + Vit D	Ca 1000 mg/d + Vit D 10 µg/d
Bolton 2012 [17]	Australia	19/18	52 wk	16.8 ± 6.6/18.1 ± 8.3	66.2 ± 4.9/66.8 ± 4.8	Combined resistance + impact training	3×/wk (center + home)	Ca + Vit D	Ca 1000 mg/d + Vit D_3_ 1000 IU/d
Basat 2013 [18]	Turkey	11/12/12	6 mo	7.6 ± 4.4/6.6 ± 4.5/7.9 ± 4.1	55.9 ± 4.9/55.6 ± 2.9/56.2 ± 4.0	Strength and high-impact groups	3×/wk, 60 min	Ca + Vit D	Ca 1200 mg/d + Vit D 800 IU/d
ElDeeb 2020 [19]	Egypt	22/21	24 wk	12.9 ± 4.5/13.3 ± 4.2	55.1 ± 4.2/57.3 ± 4.4	WBV	2×/wk, progressive 6 mo	Ca + Vit D	Ca 1200 mg/d + Vit D 800 IU/d
Sen 2020 [20]	Turkey	15/16/18	6 mo	8.2 ± 4.3/6.6 ± 4.5/7.9 ± 4.1	56.3 ± 4.8/55.6 ± 2.9/56.2 ± 4.0	WBV/high-impact exercise	3×/wk	Ca + Vit D	Ca 1500 mg/d + Vit D 880 IU/d
Iwamoto 2001 [21]	Japan	8/20	24 mo	16.3 ± 5.9/14.8 ± 6.4	65.3 ± 4.7/64.9 ± 5.7	Walking + calisthenics	≥5×/wk, 1 yr	Ca	Ca lactate 2.0 g/d + 1α(OH)Vit D_3_ 1 µg/d
Bergström 2008 [22]	Sweden	48/44	12 mo	10 ± 5	58.9 ± 4.3/59.6 ± 3.6	Brisk walking + resistance	3–5×/wk	Ca + Vit D	NA
Cai 2018 [23]	China	30/30	12 mo	≥2	52.1 ± 4.2/51.4 ± 4.9	Baduanjin	2×/day, 1 yr	Caltrate D	Ca 0.6 g/d
Zhang 2017 [24]	China	36/36	12 mo	5.9 ± 0.8/5.8 ± 0.6	53.5 ± 1.4/53.7 ± 1.1	Baduanjin	2×/day, ≥1 yr	Caltrate D	Ca 0.6 g/d
Gong 2006 [25]	China	22/22	12 mo	11 ± 4.8	61.3 ± 6.9/62.1 ± 7.0	Mountain hiking	5–7×/wk	Ca	Ca 600 mg/d
Chen 2015 [26]	China	50/50	6 mo	NA	58.7 ± 7.5/58.4 ± 6.5	Aerobic exercise	≥30 min/day	Ca + Vit D	~529 mg Ca/d + 9 µg Vit D3/d
Qin 2017 [27]	China	25/25	6 mo	NA	NA	Square dancing	5×/wk	Caltrate D	Ca 600 mg/d
Liu 2007 [28]	China	36/32	6 mo	6.8 ± 1.2	56.3 ± 2.1	Combined stretching + jogging	3–5×/wk	Caltrate D	Ca 600 mg/d

Note: WBV = whole-body vibration; Vit D = vitamin D; Ca = calcium; IU = international units; µg = microgram; g = gram; mo = month(s); wk = week(s); yr = year(s); T = intervention group; C = control group. NA = not applicable.

## Data Availability

All data generated or analyzed during this study are included in this published article (and its Supplementary Files).

## Supplementary Materials

The following supporting information can be downloaded at https://www.mdpi.com/article/10.3390/nu17243866/s1, S1: Search strategy; S2: Sensitivity analysis; S3: Meta regression; S4: Funnel plot; S5: GRADE Evidence Profile for All Outcomes.

## Abbreviations

The following abbreviations are used in this manuscript:

PMO	Postmenopausal osteoporosis
BMD	Bone mineral density
OC	Osteocalcin
P1NP	Procollagen type I *N*-terminal propeptide
CTX	*C*-terminal telopeptide of type I collagen
CNKI	National Knowledge Infrastructure
SinoMed	Chinese Biomedical Literature Database
RCTs	Randomized controlled trials
RevMan	Review Manager
MD	Mean difference
SD	Standard deviations
SMD	Standardized Mean Difference
CI	Confidence interval
WBV	Whole-body vibration
LS BMD	Lumbar spine bone mineral density
FN BMD	Femoral neck bone mineral density
GT BMD	Greater trochanter bone mineral density
Ward’s BMD	Ward’s triangle bone mineral density
TH BMD	Total hip bone mineral density
RT	Resistance training
1RM	One-repetition maximum
