# Supplementary material for: Effects of Combined Exercise and Calcium/Vitamin D Supplementation on Bone Mineral Density in Postmenopausal Women: A Systematic Review and Meta-Analysis
Source: Nutrients. 2025 Dec 11;17(24):3866. doi: 10.3390/nu17243866 (PMC12735737; doi:10.3390/nu17243866)
Supplement: Supplementary file 1 [file nutrients-17-03866-s001.zip › nutrients-4013031-supplementary.pdf]

S1: Search strategy

#1 "Osteoporosis, Postmenopausal"[Mesh] OR "postmenopausal osteoporosis"[Title/Abstract] OR "postmenopausal women"[Title/Abstract] OR "menopause"[Title/Abstract]

#2 "Bone Density"[Mesh] OR "bone mineral density"[Title/Abstract] OR BMD[Title/Abstract] OR "bone mass"[Title/Abstract] OR "bone loss"[Title/Abstract] OR "bone health"[Title/Abstract]

#3 "Exercise"[Mesh] OR "Physical Fitness"[Mesh] OR "Resistance Training"[Mesh] OR "Weight-Bearing Exercise"[Mesh] OR "Walking"[Mesh] OR "Vibration"[Mesh] OR exercise*[Title/Abstract] OR training[Title/Abstract] OR "resistance"[Title/Abstract] OR "weight-bearing"[Title/Abstract] OR "whole-body vibration"[Title/Abstract] OR "Baduanjin"[Title/Abstract] OR "Tai Chi"[Title/Abstract] OR "aerobic"[Title/Abstract] OR "physical activity"[Title/Abstract]

#4 "Calcium, Dietary"[Mesh] OR "Calcium, Dietary"[Title/Abstract] OR "Calcium supplementation"[Title/Abstract] OR "Vitamin D"[Mesh] OR "Vitamin D supplementation"[Title/Abstract] OR "calcium and vitamin D"[Title/Abstract] OR "Ca and Vit D"[Title/Abstract]

#5 #3 AND #4

#6 #1 AND #2 AND #5

#7 randomized controlled trial[Publication Type] OR controlled clinical trial[Publication Type] OR random*[Title/Abstract] OR placebo[Title/Abstract]
OR "clinical trial"[Title/Abstract] OR "RCT"[Title/Abstract]

#8 animals[Mesh] NOT humans[Mesh]

#9 #6 AND #7 NOT #8

S2: Sensitivity analysis

Figure S1. Sensitivity analysis for lumbar spine bone mineral density (LS BMD)

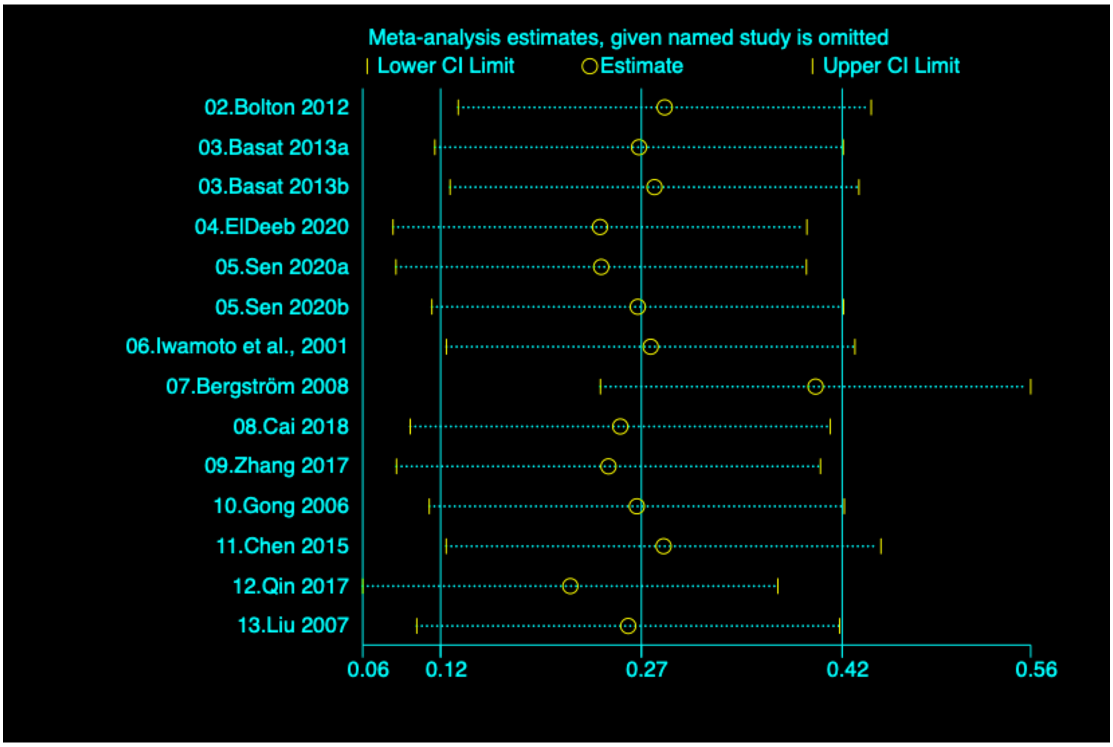

Figure S2. Sensitivity analysis for femoral neck bone mineral density (FN BMD)

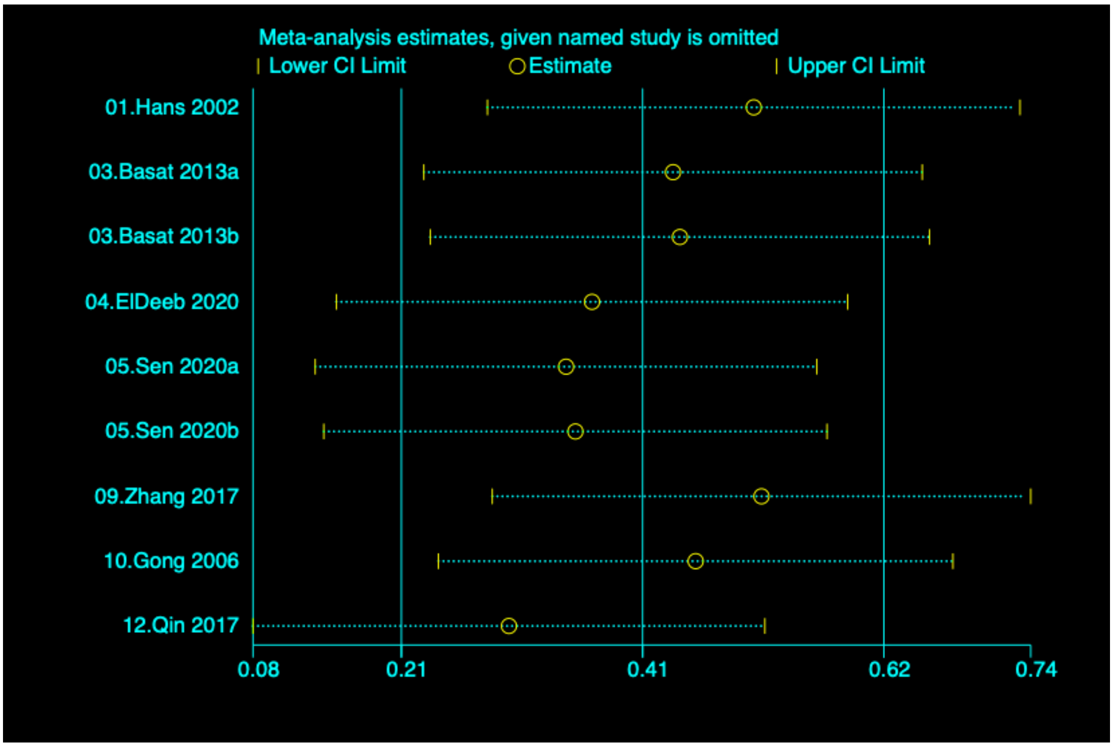

Figure S3. Sensitivity analysis for greater trochanter bone mineral density (GT BMD)

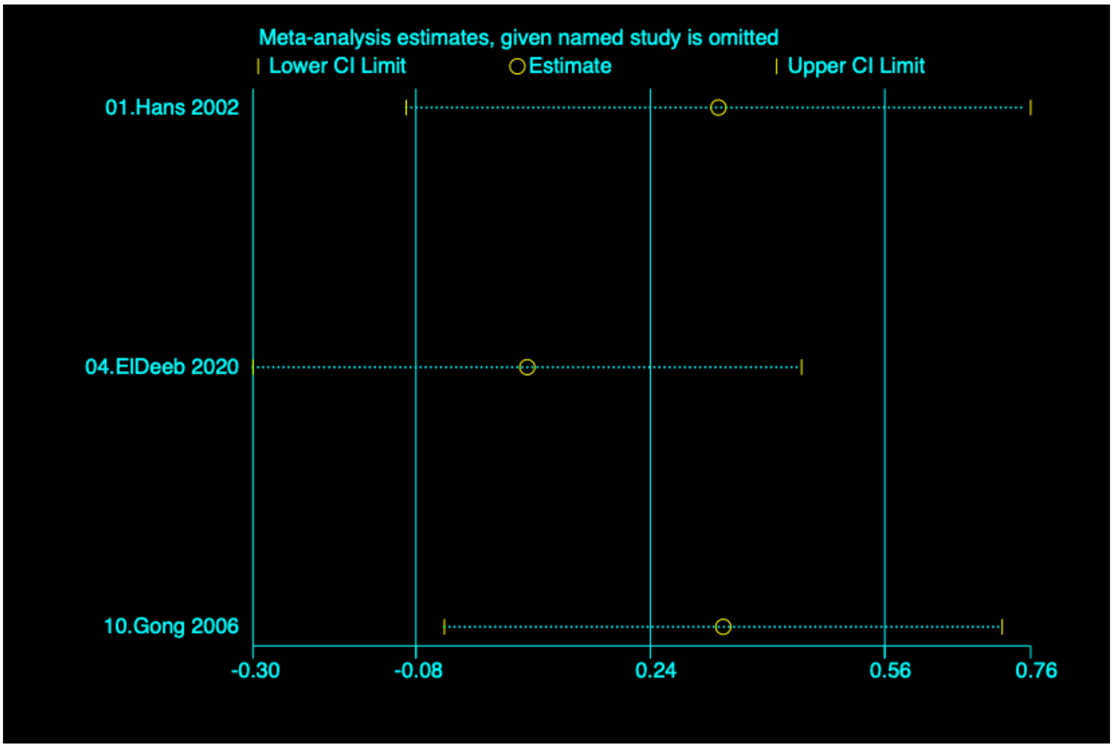

Figure S4. Sensitivity analysis for Ward’s triangle bone mineral density (Ward’s BMD)

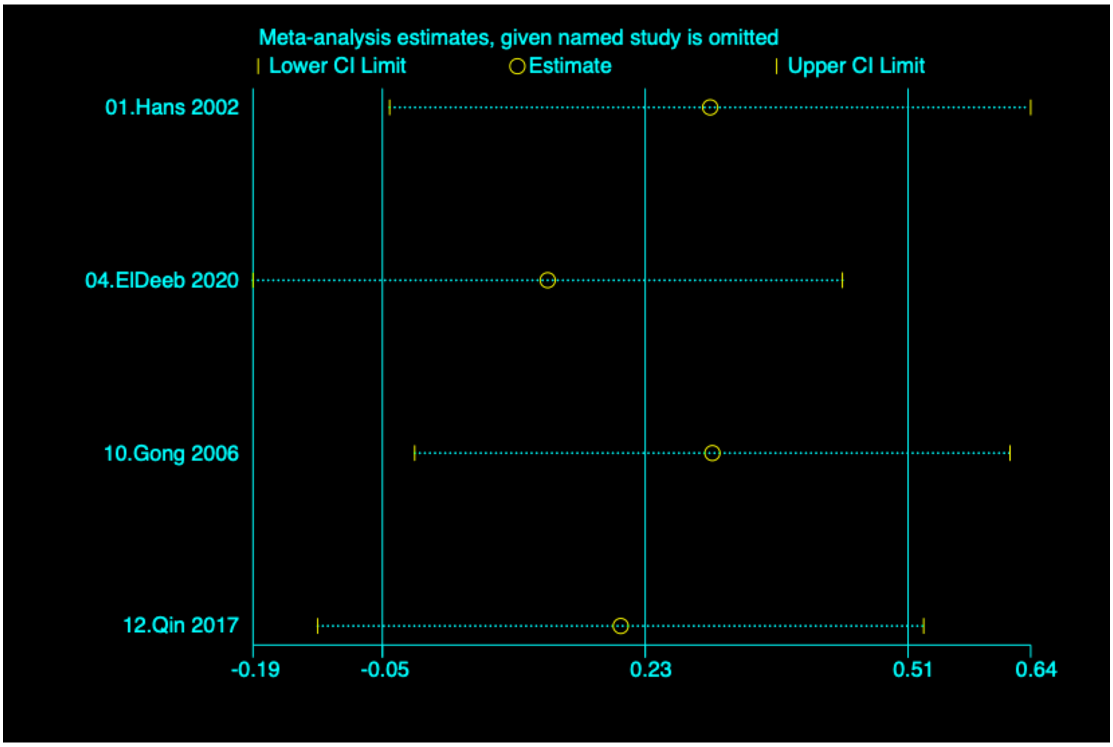

Figure S5. Sensitivity analysis for total hip bone mineral density (TH BMD)

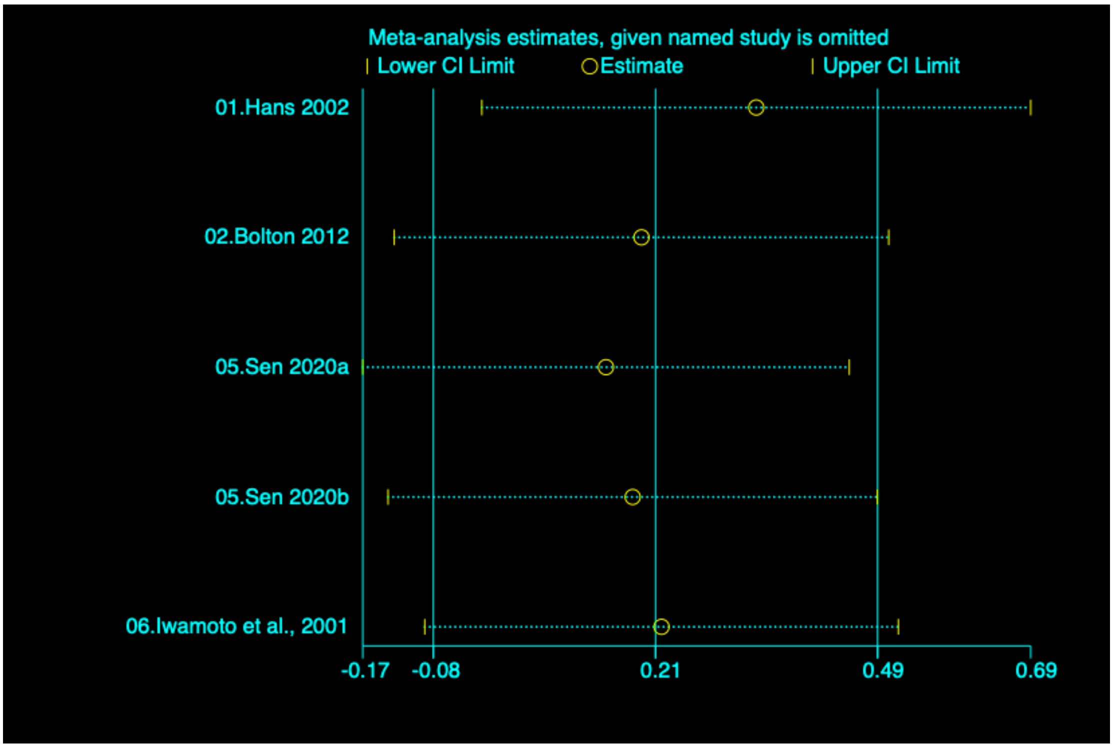

S3: Meta regression

Factors	LS BMD			FN BMD			GT BMD			Ward's BMD			TH BMD		
	Coefficient	*95% CI*	***P* value**	*Coefficient*	*95% CI*	***P* value**	*Coefficient*	*95% CI*	***P* value**	*Coefficient*	*95% CI*	***P* value**	*Coefficient*	*95% CI*	***P* value**
**Mean age (years)**	0.02	(−0.05, 0.09)	0.56	0.03	(−0.04, 0.10)	0.44	0.01	(−0.06, 0.08)	0.72	0.02	(−0.04, 0.09)	0.49	0.01	(−0.06, 0.08)	0.74
**Years since menopause**	−0.04	(−0.12, 0.04)	0.32	−0.03	(−0.11, 0.05)	0.47	−0.01	(−0.09, 0.07)	0.81	−0.02	(−0.09, 0.06)	0.65	−0.03	(−0.10, 0.05)	0.53
**Calcium/Vitamin D dosage**	0.05	(−0.03, 0.14)	0.23	0.04	(−0.04, 0.12)	0.33	0.03	(−0.05, 0.11)	0.48	0.04	(−0.04, 0.13)	0.32	0.02	(−0.06, 0.10)	0.59
**Region (Asia vs non-Asia)**	0.06	(−0.03, 0.15)	0.19	0.07	(−0.02, 0.16)	0.13	0.05	(−0.04, 0.14)	0.26	0.04	(−0.05, 0.13)	0.37	0.06	(−0.03, 0.15)	0.18

S4: Funnel plot

Figure S6. Funnel plot for publication bias assessment of lumbar spine bone mineral density (LS BMD).

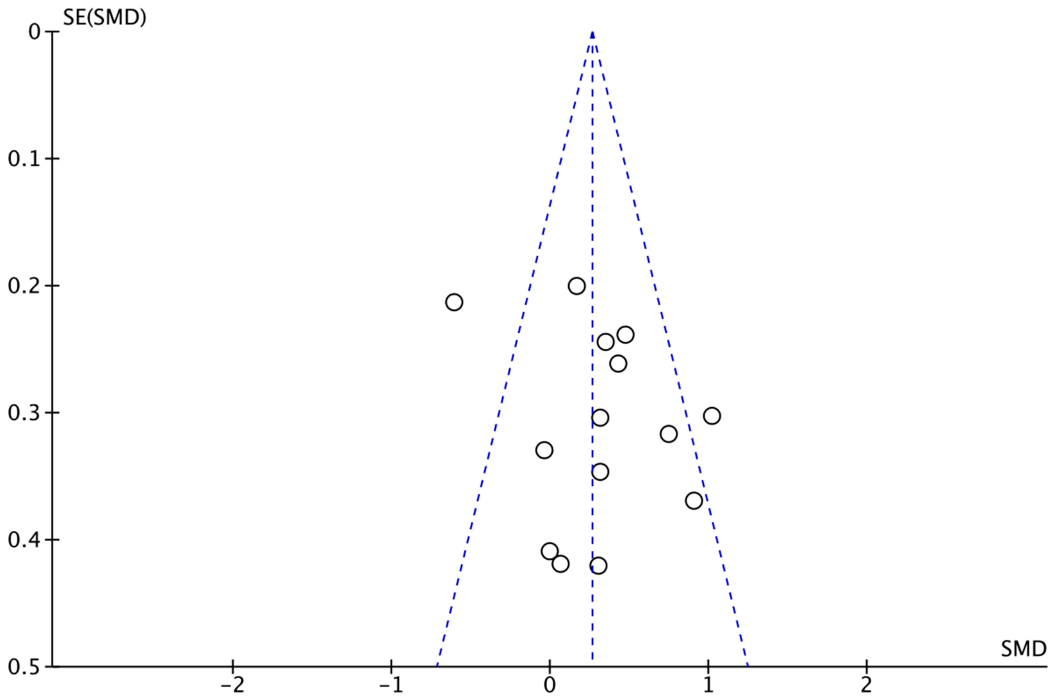

Figure S7. Funnel plot for publication bias assessment of femoral neck bone mineral density (FN BMD).

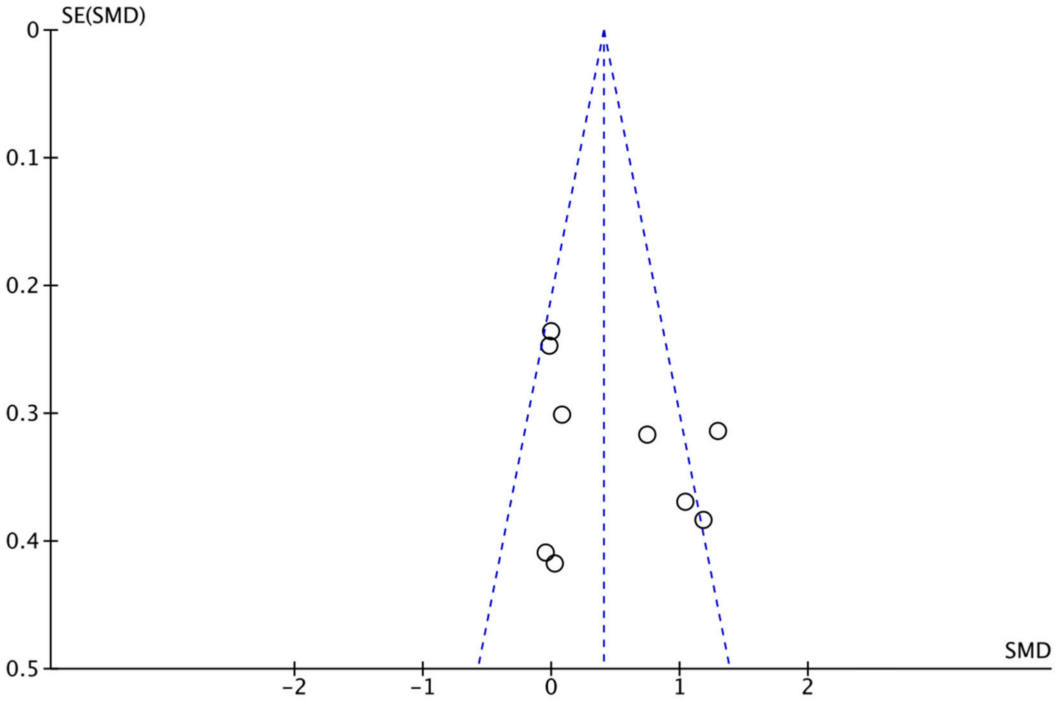

S5: GRADE Evidence Profile for All Outcomes

Certainty assessment							№ of patients		Effect		Certainty	Importance
№ of studies	Study design	Risk of bias	Inconsistency	Indirectness	Imprecision	Other considerations	BMD	placebo	Relative (95% CI)	Absolute (95% CI)		
**LS BMD**												
14	randomised trials	serious	not serious	not serious	not serious	none	350	358	-	SMD **0.31 higher** (0.06 higher to 0.55 higher)	⨁⨁⨁◯ Moderate	
**FN BMD**												
9	randomised trials	serious	not serious	not serious	not serious	none	223	186	-	SMD **0.47 higher** (0.09 higher to 0.84 higher)	⨁⨁⨁◯ Moderate	
**GT BMD**												
3	randomised trials	serious	not serious	not serious	not serious	none	108	65	-	SMD **0.24 higher** (0.08 lower to 0.56 higher)	⨁⨁⨁◯ Moderate	
**Ward's BMD**												
4	randomised trials	serious	not serious	not serious	not serious	none	133	90	-	SMD **0.22 higher** (0.05 lower to 0.5 higher)	⨁⨁⨁◯ Moderate	
**TH BMD**												
5	randomised trials	serious	not serious	not serious	not serious	none	122	96	-	SMD **0.2 higher** (0.08 lower to 0.49 higher)	⨁⨁⨁◯ Moderate	
